# Books Review

**Published:** 1871-06

**Authors:** 


### Books Review.

*Chemistry:* General, Medical and Pharmaceutical, including the Chemistry of the U. S. Pharmacopœia. A manual on the general principles of the science and their applications to medicine and pharmacy. By John Attfield, M. D., F.C.S. &c., Ph. From the second enlarged English edition. Revised by the author. Philadelphia, Henry C. Lea, 1871, 8 vo. 552 pp.

Mr. Attfield shows no small amount of well directed labor in laying before the profession engaged in pharmacy, a manual and guide of so great value. It contains " the chemistry of every substance recognised officially or in general practice as a remedial agent." He commences with the principal properties of the elements, followed by the official preparations and tests of the metallic elements, the acids and their salts; the general qualitative analysis forms the next subject, which is followed by chemistry relative to animal and vegetable substances; then follow chapters devoted to the chemical toxicology, and the examination chemically and microscopically of morbid urine, urinary sediments and calculi, and closes with quantitative analysis. The appendix contains valuable tables of tests for impurities in pharmacopœial preparations. Each subject is followed by a series of well selected questions, designed for testing the proficiency of the student as he proceeds. We cannot but recommend the woık, not only to the student of pharmacy, but also to the practising physician, who may find many items of interest and value in its pages.

---

*Wasting Disease of Children.* By EUSTACE SMITH, M. D., Lond. Second American Edition, Philadelphia. Henry C. Lea, 1871.

Of the practical value of this work we have previously had occasion to speak, so that upon the appearance of the second edition it remains only for us to note the changes which have been made. It appears, from the "advertisement" of the second edition, that "the text of the first edition has been carefully revised, inaccuracies have been corrected, and additions suggested by increased experience, have been freely introduced." Two chapters have also been introduced, and a few illustrative cases have been added. This book has a peculiar title, which would lead one to infer that it is a monograph upon "marasmus." Such however is not the case. The chronic diseases of infancy are thoroughly and wisely discussed, and the conditions of perverted nutrition incident to nearly all, are explained and their causes traced. For the general practitioner of medicine the work is invaluable; it has only to be known to be appreciated.

---

*Change of Life.* By EDWARD JOHN TILT, M. D. From the Third London Edition. Philadelphia, Lindsay & Blakiston, 1871.

This is a practical treatise on the nervous and other affections incidental to women about the menstrual decline. From what we have been able to examine the work before us, we conclude that it presents the philosophy of the diseases incident to women, at the decline of the menstrual period, in a truer and clearer manner than has before been done, and that all the advances recently made by the most careful students of nervous diseases have been truthfully incorporated into this book.

The field is truly very large, comprising the physiology, principles of pathology, and principles of treatment and hygiene at the change of life. Diseases of the brain and ganglionic, nervous system, with the various neuralgic affections, together with diseases of the reproductive and gastro-intestinal organs, and all other affections occurring at the change of life, are embodied in the work. We think it cannot be too carefully studied or too highly prized.

*Insanity and its Treatment.* Lectures on the treatment, Medical and Legal, of Insane Patients. By G. FIELDING BLANDFORD, M. D., Oxon. With summary of laws in force in the United States on the confinement of the Insane, by ISAAC RAY, M. D.

We are happy to announce the publication of this book, for it has, for a long time, been greatly needed by the profession. Vague, unsettled and even erroneous views of insanity prevail, to a great extent, in the profession, and a systematic work presenting the recent and more scientific views as to the nature, causes, morbid changes and treatment of insanity will be received by the general practitioners of medicine with great satisfaction.

This work is evidently arranged with special reference to the wants of the general practitioner, and we know of no work which at present equals it in this respect. It consists of twenty lectures, which were given at St. George's Hospital, London, and condensed, constitutes the work before us.

We regret that space will not allow us to speak in detail of the teaching of the work. It must be read and studied to be appreciated, and we earnestly recommend it as a standard authority on the subject, to be in the library of all physicians.

---

*The Medical Section of the work of N. P. Daubeveyer. A Vade-mecum for the use of Invalids and tourists visiting Carlsbad.* By Dr. GANS, resident physician. Carlsbad, 1871.

This little pamphlet contains the temperature, chemical constituents and action of the water at the springs at Carlsbad, and for the benefit of those who may visit Carlsbad, we will give them in detail. The spring of Marktbrunn attains 390 Reaumur, while that of the Sprudel reaches 590 R. Their chemical constituents are: Sulphate of soda, chloride of sodium, carbonate of soda and carbonic acid. The author speaks highly of the beneficial effects of these ingredient. "Carlsbad Salts," containing sulphate of soda, chloride of sodium and carbonate of soda, which are used as a safe and effective purgative, may be obtained by evoporating the water of the Sprudel.

---

*Annual Report of Commissioners of Quarantine.*

The above pamphlet contains interesting particulars of the proceedings of the Commissioner, and a statement of the condition of the establishment for the past year. There is also annexed the "Annual Report" of Dr. Carnochan, Health officer of the port of New York, containing some interesting mention of contagious and infectious diseases met with during the year past.

---

*Dactylitis Syphilitica, with observations on Syphilitic Lesion of the Joints.* By R. W. TAYLOR, M. D., Surgeon to New York Dispensary, &c.

This is a reprint from the American Journal of Syphilography and Dermatology, of an article which has attracted some considerable notice among medical Journals. The author treats his subject thoroughly, illustrating his views with several wood cuts. This paper will repay a careful perusal.

*Uterine Catarrh frequently the cause of Sterility.* By H. E. Gantillon, M. D.

This work was originally written in French, and the Doctor therefore apologises for any gallicisms and inaccuracies which may have escaped him in the hurry of translation. The Pamphlet covers the entire ground, leaving out none of the essential points, and gives us his "New Treatment" for its cure by "Intra-Uterine Injections," proving its efficacy by numerous clinical cases.

---

*The Study of Dermatology.* By Louis A. Duhring, M. D., Physician to Dispensary for Skin Diseases, Philadelphia.

Reprint of Journal of Syphilography and Dermatology. A criticism of considerable merit on a subject which offers a large field for the critics pen. The author gives us the views which the German, French and English schools maintain on this subject.

---

*Hæmatoma Auris.* By E. R. Hun, M. D.

A paper having for its central idea, "The phenomena and Pathology of Hæmatoma in relation to Mental Derangement." Dr. Hun is connected with the New York State Lunatic Asylum, and having had the best possible opportunities for developing his views, is prepared to speak from original observations. Before giving an account of individual cases, the doctor treats us to a general outline of the appearance, pathology, &c., of the disease, and then gives us his clinical observations, which number some twenty or more cases, five of which are photographically illustrated.

